# Supervised Learning Computer Vision Benchmark for Snake Species Identification From Photographs: Implications for Herpetology and Global Health

**DOI:** 10.3389/frai.2021.582110

**Published:** 2021-04-20

**Authors:** Andrew M. Durso, Gokula Krishnan Moorthy, Sharada P. Mohanty, Isabelle Bolon, Marcel Salathé, Rafael Ruiz de Castañeda

**Affiliations:** ^1^ Department of Biological Sciences, Florida Gulf Coast University, Ft. Myers, FL, United States; ^2^ Institute of Global Health, Faculty of Medicine, University of Geneva, Geneva, Switzerland; ^3^ Eloop Mobility Solutions, Chennai, India; ^4^ AICrowd, Lausanne, Switzerland; ^5^ Digital Epidemiology Laboratory, École Polytechnique Fédérale de Lausanne, Geneva, Switzerland

**Keywords:** fine-grained image classification, crowd-sourcing, reptiles, epidemiology, biodiversity

## Abstract

We trained a computer vision algorithm to identify 45 species of snakes from photos and compared its performance to that of humans. Both human and algorithm performance is substantially better than randomly guessing (null probability of guessing correctly given 45 classes = 2.2%). Some species (e.g., *Boa constrictor*) are routinely identified with ease by both algorithm and humans, whereas other groups of species (e.g., uniform green snakes, blotched brown snakes) are routinely confused. A species complex with largely molecular species delimitation (North American ratsnakes) was the most challenging for computer vision. Humans had an edge at identifying images of poor quality or with visual artifacts. With future improvement, computer vision could play a larger role in snakebite epidemiology, particularly when combined with information about geographic location and input from human experts.

## Introduction

Snake identification to the species level is challenging for the majority of people (Henke et al., 2019; Wolfe et al., 2020), including healthcare providers who may need to identify snakes (Bolon et al., 2020) involved in the ∼5 million snakebite cases that take place annually worldwide (Williams et al., 2019). The current gold standard in the clinical management of snakebite is identification by an expert (usually a herpetologist; Bolon et al., 2020; Warrell, 2016), but experts are limited in their number, geographic distribution, and availability. Snakes are never identified in nearly 50% of snakebite cases globally (Bolon et al., 2020) and even in developed countries with detailed record keeping, species-level identification of snakes in snakebite cases could be improved. For instance, only 5% of snake bites in the United States from 2001 to 2005 were reported at the species level and 30% of bites were from totally unknown snakes (Langley, 2008); more recently, only 45% of snake bites in the United States from 2013 to 2015 were identified to the species level (Ruha et al., 2017).

Computer vision can make an impact by speeding up the process of suggesting an identification to a healthcare provider or other person in need of snake identification. Once just a dream (Gaston and O’Neill, 2004), AI-based identification exists for other groups of organisms (e.g., plants, fishes, insects, birds; Hernández-Serna and Jiménez-Segura, 2014; Barry 2016; Seeland et al., 2019; Wäldchen et al., 2018; Wäldchen and Mäder, 2018) but all applications for snakes have so far been quite limited in scope (James et al., 2014; Amir et al., 2016; James, 2017; Joshi et al., 2018; Joshi et al., 2019; Rusli et al., 2019; Patel et al., 2020), being focused on only a few species or using only high-quality training images from a limited number of individuals often taken under captive conditions that do not reflect the variation in quality and background that characterize photos taken by amateurs in the wild.

Performance of computer vision algorithms depends on the quality of the training data as well as on the learning mechanism. Generating realistic and unbiased training and testing data is a major challenge for most computer vision applications, especially for species of animals, which have greater intraclass (inter-individual) variation than manufactured objects such as street signs or license plates (Stallkamp et al., 2012). Although models that are pre-trained on generic publicly available image datasets (e.g., ImageNet, Object Net) can meet or exceed state-of-the-art performance on several vision benchmarks after fine-tuning on just a few samples (Barbu et al., 2019), biodiversity is so vast that targeted labeled training datasets where each species represents one class must be used to achieve desired performance benchmarks.

An ideal diagnostic tool for snakebite would support healthcare providers in reporting the taxonomic identity of biting snakes, which would vastly improve articulation of taxonomic names of snake species with medical records of bite symptoms and improve snakebite epidemiology data, responses to specific treatment, and antivenom efficacy [see also Garg et al. (2019) for discussion of this problem with genetic resources]. In certain cases, improved snake identification capacity could also aid in clinical management; for example, asymptomatic patients with bites from non-venomous snakes could be released sooner, and knowing which species of medically important venomous snake (which make up ∼20% of all snake species) is involved could allow healthcare providers to select among a possible diversity of monovalent or polyvalent antivenoms and anticipate the appearance of particular symptoms. Currently, the approach to all these diagnostics is primarily syndromic—in the absence of herpetological expertise, many healthcare providers await the appearance of symptoms and then treat based on a diagnosis made from these symptoms.

Our goal was to develop a computer vision algorithm to identify species of snakes to support healthcare providers and other health professionals and neglected communities affected by snakebite (Ruiz De Castaneda et al., 2019). Our project is a use case of the ITU-WHO Focus Group on “AI for Health” (Wiegand et al., 2019), with the aim of creating “a rigorous, standardised evaluation framework” in order to promote the responsible adoption of algorithmic decision-making tools in health. In our opinion, an ideal benchmark in snake identification would be >99% top-1 identification accuracy to the species level from a single photo of low quality.

## Methods

### Classes and Training Dataset

Although there are >3,700 species of snakes worldwide (Uetz et al., 2020), 600–800 of which are medically important (Uetz et al., 2020), we chose to initially focus on 45 of the most well-represented species, each with ≥500 photos per species (i.e., per class). We gathered a total of 82,601 images from open online citizen science biodiversity platforms (iNaturalist, HerpMapper) and photo sharing sites (Flickr). The photos were labeled by users of these platforms; either by the user who uploaded the photo (Flickr, HerpMapper) or through a consensus reached by users who viewed the photo and submitted an identification (iNaturalist; see Hochmair et al., 2020 for a synopsis). The data from iNaturalist were collected Oct 24, 2018 using the iNaturalist export tool (https://www.inaturalist.org/observations/export) with the parameters quality_grade=research&identifications=any&captive=false&taxon_id=85553. The HerpMapper data were provided via a partner request on 19 Sept 2018 (see https:// www.herpmapper.org/data-request). The Flickr data were collected Nov 5, 2018 using a python script (https://github.com/cam4ani/snakes/blob/master/get_flickr_data.ipynb). The full training dataset can be accessed at (https://datasets.aicrowd.com/aws-eu-central1/aicrowd-static/datasets/snake-species-identification-challenge/train.tar.gz)

In order to improve the accuracy of labels used for image classification, we employed several best practices. For the iNaturalist data, only “research grade” observations, which require a community-supported and agreed-upon identification at the species taxonomic rank, were used. Additionally, we selected a subset (N = 336) of these images for additional label validation by human experts (Durso et al., 2021) and found that just five (1.5%) were misidentified (these have been corrected on iNaturalist). HerpMapper is used primarily by experienced enthusiasts with a lot of experience in snake identification; we found no misidentifications in the subset (N = 200) we examined. Finally, we used only scientific names in our Flickr search to target photographers with a more serious interest in biodiversity. We found no misidentifications in the subset (N = 63) we examined, although one image showed only the habitat without an actual snake. Subset sample sizes were chosen to represent 0.5% of the total dataset from each source. Human experts (N = 250) were recruited via social media, targeting Facebook groups that specialize in snake identification (Durso et al., 2021) as well as by email or private messages over iNaturalist to top identifiers. Because of privacy issues, we were unable to collect demographic information about this community of experts. Their accuracy in aggregate was 43% at the species level, 56% at the genus level, and 75% at the family level, with important variation among individuals, snake species, and global region (Durso et al., 2021).

**FIGURE 1:**
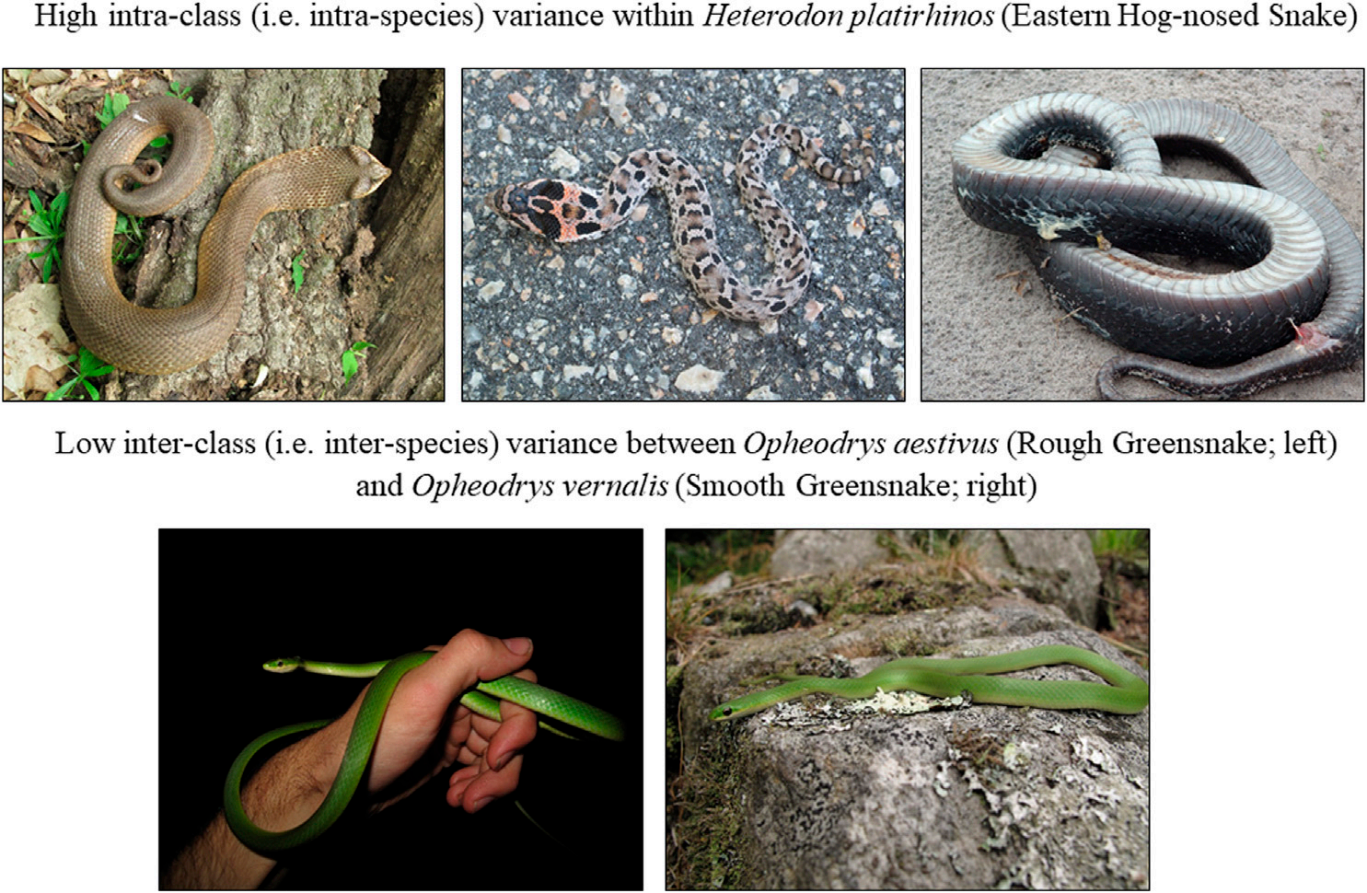
Examples of high intra-class (i.e., intra-species) and low inter-class (i.e., inter-species) variance among snake images. All photos by Andrew M. Durso (CC-BY).

The distribution of photos among classes was unequal: the class with the most photos had 11,092, while the class with the fewest photos had 517. Some species classes have high intraclass variance (Figure 1), due to geographic and ontogenetic variation (e.g., Manier, 2004), and to color and pattern polymorphism (e.g., Shannon and Humphery, 1963; Cox et al., 2012), and others have very low interclass variance (Figure 1), due to morphological similarity among closely related species as well as inter-species and even inter-family mimicry (e.g., Sweet, 1985; Akcali and Pfennig, 2017). The 45 species we used are found largely in North America (42 species in 19 genera; see Appendix I for a full list), with two Eurasian species (*Hierophis viridiflavus* and *Natrix natrix*) and one Central/South American species (*Boa constrictor*). An interactive version of Figure 1 is available at https://chart-studio.plotly.com/∼amdurso/1/#/.

### Algorithm Development Challenge

We used the platform AICrowd, which takes a collaborative approach to the development of algorithms, by inviting data science experts and enthusiasts to collaboratively solve real-world problems by participating in challenges in which the solutions are automatically evaluated in real-time. On January 21, 2019, we launched a “Snake Species Identification Challenge”. The first round lasted until May 31, 2019, and the second round (during which live code collection was implemented) until July 31, 2019. We offered prizes as incentives for the best algorithms submitted (a travel grant and co-authorship on this manuscript). A total of 24 participants made 356 submissions, resulting in five algorithms with an F1 ≥ 0.75 and a top score of F1 = 0.861 with a log-loss = 0.53.

### Test Datasets

We evaluated the identification accuracy of the submitted algorithms using two test datasets (TD2 and TD3), both distinct from the training dataset described above and its subset (TD1) used for pre-submission testing by the challenge participant (Table 1). One (TD2) was made up of 42,688 photos of the same 45 species of snakes submitted to iNaturalist between January 1, 2019 and September 2, 2019 (after the beginning of our challenge; range 23–6,228 photos per class). The other (TD3) was made up of 248 undisclosed images from 27 classes (1–10 images per class) that were collected from private individuals. Many of the images in this dataset were purposefully chosen to be as difficult to identify as possible—e.g., low resolution, out of focus, with the snake filling only a small part of the frame, and/or obscured by vegetation. The identity of species in these images was confirmed by a herpetologist (A. Durso) and they were used in a citizen science challenge where they were presented to participants recruited from online snake identification communities (largely Facebook snake identification groups and iNaturalist) who suggested species identifications, resulting in 68–157 labels of 5–47 classes per image (Durso et al., 2021). We further subdivided TD3 to take all 45 classes into account (TD3a; for fairer comparison with human labeling, because humans were allowed to choose any of the >3,700 snake species classes from a list), and to evaluate only the 27 classes in common (TD3b; for fairer comparison with TD2).

**TABLE 1:** Summary of three test datasets used to evaluate identification accuracy of top algorithm. Performance of humans on TD3a yielded F1 = 0.76, accuracy = 68%, error = 32%, and on TD3b F1 = 0.79, accuracy = 79%, error = 21%. *The winning F1 high score of 0.861 reported above comes from a different randomly generated subset, not reported here in detail.

Test dataset	TD1	TD2	TD3a	TD3b
# of images	16,483	42,688	248	
# of classes	45	45	27	
# of classes used to compute F1	45	45	45	27
Minimum # of images per class	94	23	1	
Maximum # of images per class	2,226	6,228	10	
F1 score	0.83*	0.83	0.53	0.73
Log-loss	0.49	0.66	1.19	1.03
Accuracy	87%	84%	73%	72%
Error	13%	16%	27%	28%
Dataset description	Subset of training dataset. Used for pre-submission testing by winning challenge participant	Photos submitted to iNaturalist after the beginning of our challenge	Photos collected from private individuals and social media and labeled by human experts (71–156 labels per image)	
			When all 45 classes were taken into account (for fairer comparison with human labeling)	When only 27 classes were taken into account (for fairer comparison with TD1 and TD2)

### Winning Algorithm

**FIGURE 2:**
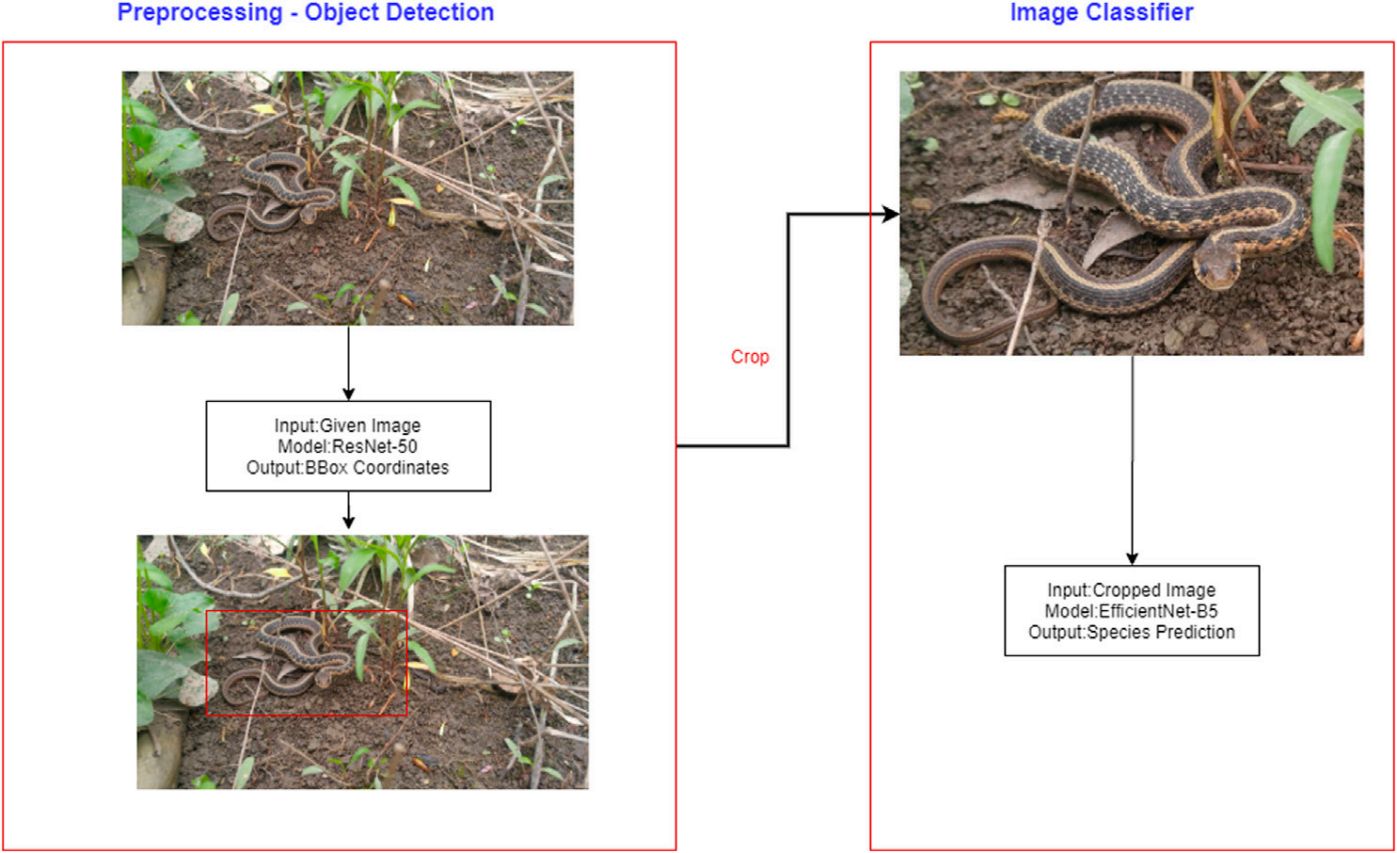
Diagram of overall pipeline including both object detection and classification.

Applying large, deep convolutional neural networks for image classification is a well-studied problem (Krizhevsky et al., 2012; Huang et al., 2017; He et al., 2019). The top algorithm made use of incremental learning in neural networks and incorporated elements of EfficientNet from Google Brain (Tan and Le, 2019), a pre-trained network from ILSRVC (Russakovsky et al., 2015), discriminative learning, cyclic learning rates and automated image object detection (Figure 2).

As shown by Kornblith et al. (2019), models trained on a standard data distribution generalize better than the models trained from scratch. The ImageNet Large Scale Visual Recognition Competition (ILSVRC) dataset is the most widely used dataset for benchmarking image classifiers, comprising 1.2 million images classified into 1,000 different classes. The winning solution applied incremental learning on a pre-trained EfficientNet network. Specifically, this involved retaining what the model has learned from the ILSVRC dataset and performing incremental learning on the snake species domain. The final layer from the pretrained network was removed and replaced with a domain specific head, a fully connected layer of size 45, each representing the probability of the snakes being in a particular class. Discriminative learning strategy was also used to train the network. Specifically, different layers of the network are responsible for capturing different types of information (Yosinski et al., 2014) and discriminative learning allows us to set the rate at which different components of the network learn. The initial layers are trained at much lower learning rates to inhibit the loss of learned information while the final layers are trained at higher learning rates. A general update to model parameters ⍵ at time step t looks like:

$$\omega_{t}=\;\omega_{t-1}-\lambda .\Delta_{\omega }J\left(\omega \right)$$

where *λ* denotes the learning rate and Δ*ω*J(*ω*) denotes the gradient with respect to the model’s objective function. In discriminative learning, the model is split into N components, {*ω* ^1^, *ω* ^2^,…*ω* ^N^} where *ω* ^n^ contains the layers of the nth component of the model. Each component can have any number of layers. An update to model parameters then becomes:

$$\omega_{t}^{n}=\;\omega_{t-1}^{n}-\lambda^{n}.\Delta_{\omega }J\left(\omega \right)$$

where *λ* ^n^ denotes the learning rate of the *n*th component of the model.

In the first attempt, an image classifier was trained using progressive resizing, starting with Densenet121 (Huang et al., 2017) architecture with a modified focal loss function (Lin et al., 2017) for multi-class image classification and resizing image sizes from (256,256) → (384,384) → (512,512) → (768,768) → (1024,1024). Using this as an initial method, the scores plateaued at F1 ∼0.67. As an alternative, a new image classifier was trained from scratch using Resnet152 with modifications as suggested by Bag of tricks (He et al., 2019) called XResnet152 (152 indicating the number of layers). This time, the scores plateaued at ∼0.75.

Adding a preprocessing pipeline to predict the four coordinates of the corners of the box bounding the snake itself (which may be any size and shape and in any orientation within the image, against any background) and crop the images, as well as handling orientation variance, was accomplished by annotating 30–32 images from the training dataset from each species category using an annotation tool called sloth (https://github.com/cvhciKIT/sloth/). Pipelining preprocessing and training with XResnet architecture together increases the accuracy to 0.78–0.79.

**FIGURE 3:**
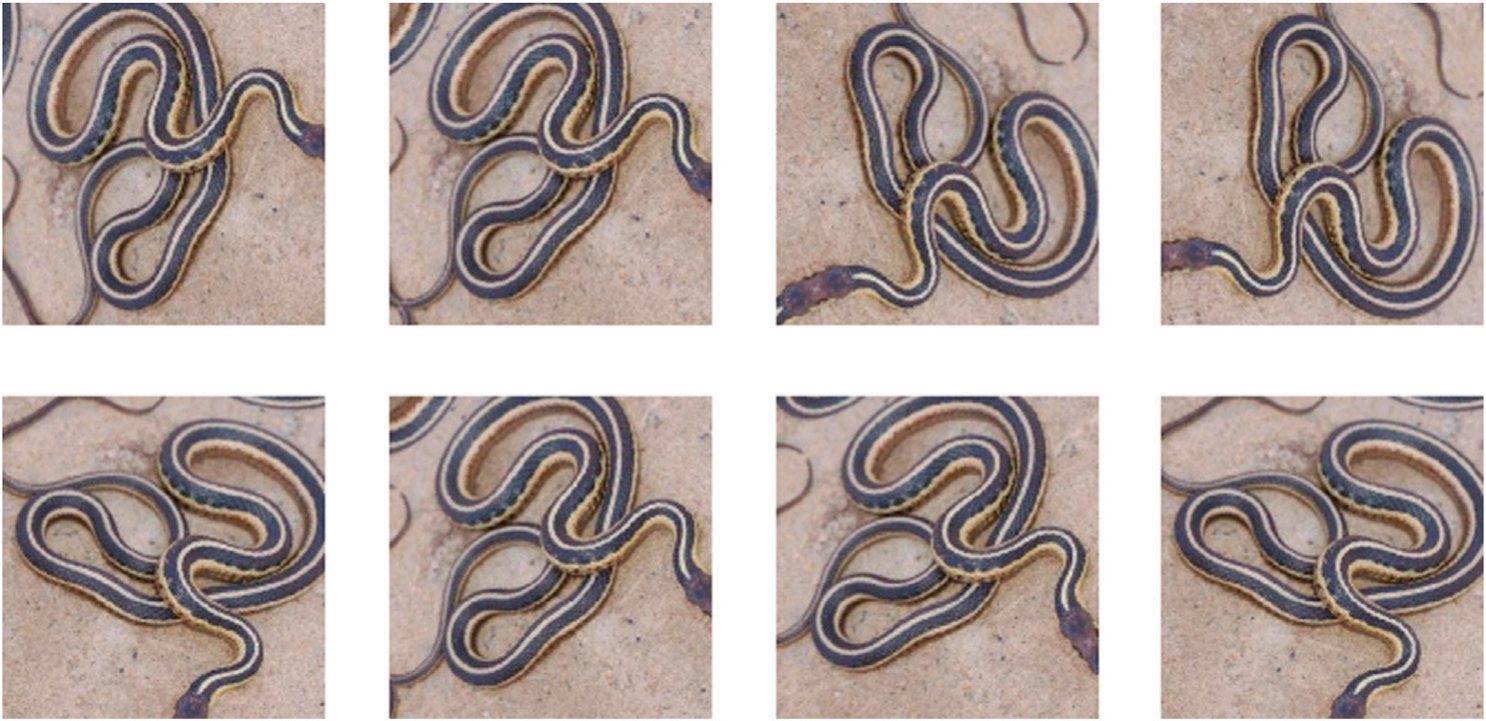
Rotation augmentation of a sample image.

Finally, a pre-trained EfficientNet (Tan and Le, 2019), which balances the depth of the architecture, the width of the architecture [Mobile Inverted Bottleneck Convolutional block (Sandler et al., 2018) with Swish activation function (Ramachandran et al., 2017)], image resolution and uses appropriate drop-outs, was fine-tuned using preprocessed images. Our winning algorithm carefully tuned hyper-parameters for EfficientNetB0 (Supplementary Figure S1) and used the exact same parameters for EfficientNetB5 (Supplementary Figure S2), although it is expected that higher accuracy is possible using the more time-intensive checkpoints/pretrained weights for B6 and B7 and by fine-tuning EfficientNetB6/B7 pre-trained on ImageNet. Some of the best hyperparameters required to train the EfficientNet include: 1) taking off the final layer of EfficientNetB5 and adding a single layer of size 45 (fastai adds an additional layer, which was not optimal for this case); 2) using the LabelSmoothingCrossEntropy Loss function; 3) using RMSProp optimizer with centered = True; 4) setting bn_wd = false, no batchnorm weight decay; 5) training with discriminative learning; 6) using resize method as SQUISH and not CROP; 7) no mixup augmentation; 8) use rotation augmentation (rotating the training image from −90 to +90 with a probability of 0.8) as shown in Figure 3; and 9) training on 95% of the dataset instead of the 80–20 split.

**FIGURE 4:**
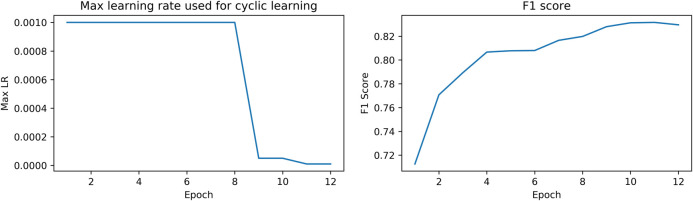
Max learning rate schedule and corresponding F1 score.

The network was trained on a single Tesla V100 GPU with a batch size of 8 for 12 epochs. The learning rate schedule is shown in Figure 4. Each epoch took approximately 55 min for completion. Other approaches that were tried but did not work include building a genus classifier to predict the snake genus and then the snake species within genus, focal loss, a weighted sampler/oversampling and batch accumulation (updates happen every n epochs, where n > 1).

The algorithm and readme are available at https://github.com/GokulEpiphany/contests-final-code/tree/master/aicrowd-snake-species.

## Results and Discussion

### First Test Dataset (TD1)

**FIGURE 5:**
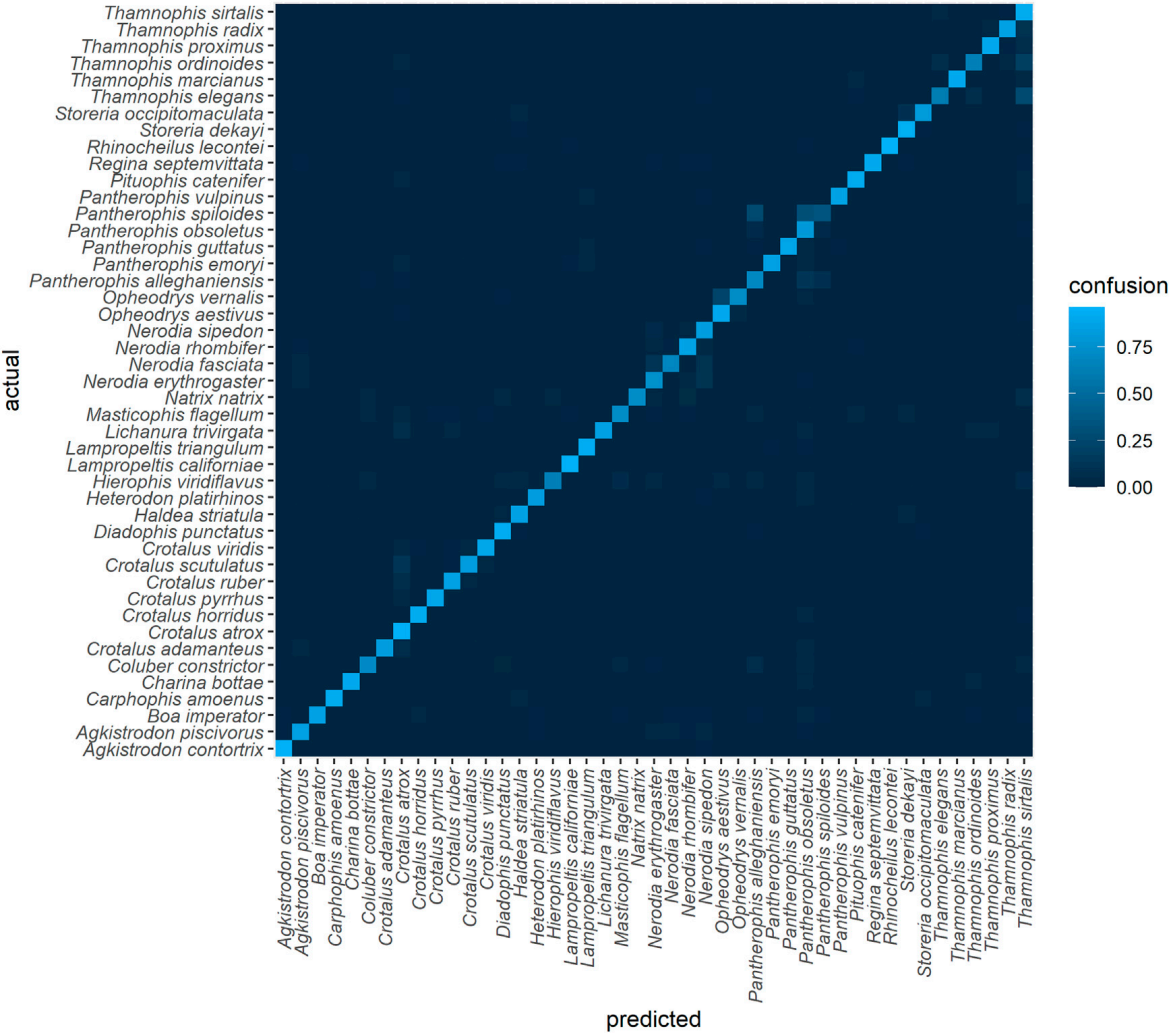
Confusion matrix for the top algorithm, using TD1 and an 80-20 split (subset of training data). The final model was trained on a 95-5 split. For an interactive version see https://chart-studio.plotly.com/~amdurso/1/#/.

A confusion matrix generated using TD1 (a withheld subset of the training data) is shown in Figure 5. Species classification accuracy ranged from 96% for *Rhinocheilus lecontei* to 35% for *Pantherophis spiloides*. Interclass confusion was 0 for 1332/2025 (66%) of class pairs. F1 for this dataset was 0.83 and log-loss was 0.49 (Table 1). Relatively high confusion remains between some similar species pairs. In particular, the three putative species of the *Pantherophis obsoletus* (North American ratsnake) complex (Burbrink, 2001) were frequently confused (Table 2).

**TABLE 2:** Confusion among putative species of the North American Ratsnake (*Pantherophis obsoletus*) complex (Burbrink, 2001) in TD1 and TD2 (the three putative species were combined in TD3). The identity of species in training and testing data was done exclusively from photos, taking into account the geographic location but not information from scale counts or DNA.

Correct ID of testing image	ID suggested by algorithm	Percent confusion in TD1	Percent confusion in TD2
*P. spiloides*	*P. obsoletus*	0.31	0.11
*P. spiloides*	*P. alleghaniensis*	0.25	0.08
*P. alleghaniensis*	*P. obsoletus*	0.13	0.07
*P. alleghaniensis*	*P. spiloides*	0.08	0.20
*P. obsoletus*	*P. alleghaniensis*	0.05	0.10
*P. obsoletus*	*P. spiloides*	0.04	0.13

Among species that are uncontroversially delimited, there were seven species pairs with confusion >10% in TD1 (max = 26% for *Thamnophis elegans* as *Thamnophis sirtalis*; Table 3). The highest confusion between species in different genera was *Natrix natrix* as *Thamnophis sirtalis* (7%). Although these genera are found in different hemispheres, confusion among co-occurring genera exceeded 5% for *Coluber constrictor* as *Pantherophis alleghaniensis* (6%) and for *Lichanura trivirgata* as *Crotalus atrox* (5%). The last is particularly troubling because *Lichanura trivirgata* is a harmless boid whereas *Crotalus atrox* is a large, potentially dangerous rattlesnake; these species co-occur in the Sonoran Desert and are probably frequently photographed against similar backgrounds (Rorabaugh, 2008). Other frequently confused intergeneric and intercontinental species pairs were *Hierophis viridiflavus* (as *Thamnophis sirtalis* and as *Masticophis flagellum*; both 5%) and *Natrix natrix* (as *Nerodia rhombifer*; 5%).

**TABLE 3:** Confusion among species that are uncontroversially delimited, showing only pairs with confusion >10% in TD1, TD2, or TD3 (by algorithm or humans). MIVS = medically important venomous snakes.

Correct ID	Given ID	TD1	TD2	TD3	Humans-all	Humans-45	Humans-27 (with geo)	Geo-overlap	MIVS
*Thamnophis elegans*	*Thamnophis sirtalis*	0.26		0.18	0.10	0.06	0.11	Y	Neither
*Opheodrys vernalis*	*Opheodrys aestivus*	0.23	0.08	0.33	0.24	0.13	0.24	Y	Neither
*Thamnophis ordinoides*	*Thamnophis sirtalis*	0.19			NA	NA	NA	Y	Neither
*Crotalus scutulatus*	*Crotalus atrox*	0.13			0.22	0.14	NA	Y	Both
*Nerodia fasciata*	*Nerodia erythrogaster*	0.11	0.07	0.25	0.04	0.02	0.02	Y	Neither
*Nerodia erythrogaster*	*Nerodia sipedon*	0.10			0.19	0.10	0.15	Y	Neither
*Storeria occipitomaculata*	*Storeria dekayi*	0.10		0.18	0.22	0.12	0.06	Y	Neither
*Nerodia sipedon*	*Nerodia erythrogaster*		0.17		0.02	0.01	0.02	Y	Neither
*Thamnophis sirtalis*	*Thamnophis radix*		0.15		0.10	0.05	NA	Y	Neither
*Pituophis catenifer*	*Lichanura trivirgata*		0.14		0.00	0.00	NA	Y	Neither
*Thamnophis sirtalis*	*Thamnophis elegans*		0.12		0.06	0.03	0.12	Y	Neither
*Opheodrys aestivus*	*Opheodrys vernalis*		0.11		0.19	0.10	0.24	Y	Neither
*Heterodon platirhinos*	*Nerodia erythrogaster*			0.25	0.01	0.00	0.00	Y	Neither
*Storeria occipitomaculata*	*Nerodia erythrogaster*			0.25	0.00	0.00	0.00	Y	Neither
*Charina bottae*	*Haldea striatula*			0.22	0.01	0.00	0.01	N	Neither
*Agkistrodon contortrix*	*Thamnophis elegans*			0.17	0.00	0.00	0.00	N	False negative
*Agkistrodon contortrix*	*Agkistrodon piscivorus*			0.14	0.04	0.02	0.11	Y	Both
*Pantherophis obsoletus*	*Agkistrodon piscivorus*			0.14	0.02	0.01	<0.01	Y	False negative
*Haldea striatula*	*Diadophis punctatus*			0.14	0.16	0.10	0.02	Y	Neither
*Agkistrodon piscivorus*	*Heterodon platirhinos*			0.14	<0.01	0.00	0.03	Y	False negative
*Storeria dekayi*	*Masticophis flagellum*			0.14	0.01	0.01	0.00	Y	Neither
*Heterodon platirhinos*	*Pantherophis obsoletus*			0.12	0.03	0.02	<0.01	Y	Neither
*Storeria dekayi*	*Haldea striatula*			0.11	0.02	0.01	0.04	Y	Neither
*Crotalus horridus*	*Agkistrodon contortrix*			0.10	0.01	0.00	<0.01	Y	Both
*Haldea striatula*	*Agkistrodon contortrix*			0.10	0.00	0.00	0.00	Y	False positive
*Crotalus scutulatus*	*Crotalus adamanteus*			0.10	0.04	0.03	0.01	N	Both
*Pantherophis guttatus*	*Crotalus horridus*			0.10	0.00	0.00	<0.01	Y	False positive
*Pituophis catenifer*	*Crotalus horridus*			0.10	0.01	0.00	<0.01	Y (barely)	False negative
*Agkistrodon piscivorus*	*Pituophis catenifer*			0.10	0.00	0.00	<0.01	Y (barely)	False negative
*Masticophis flagellum*	*Pituophis catenifer*			0.10	0.01	0.00	0.02	Y	Neither
*Pantherophis guttatus*	*Pituophis catenifer*			0.10	0.00	0.00	0.01	N	Neither
*Thamnophis radix*	*Thamnophis sirtalis*				0.23			Y	Neither
*Nerodia erythrogaster*	*Nerodia fasciata*				0.19		0.33	Y	Neither
*Lampropeltis triangulum*	*Pantherophis guttatus*				0.17			Y	Neither
*Nerodia erythrogaster*	*Agkistrodon piscivorus*				0.15			Y	False positive
*Agkistrodon piscivorus*	*Agkistrodon contortrix*				0.13			Y	Both
*Hierophis viridiflavus*	*Natrix natrix*				0.13			Y	Neither
*Nerodia sipedon*	*Nerodia fasciata*				0.11		0.11	Y	Neither
*Storeria dekayi*	*Storeria occipitomaculata*						0.19	Y	Neither
*Nerodia fasciata*	*Nerodia sipedon*						0.15	Y	Neither
*Pantherophis guttatus*	*Lampropeltis triangulum*						0.14	Y	Neither
*Natrix natrix*	*Hierophis viridiflavus*						0.14	Y	Neither
*Diadophis punctatus*	*Haldea striatula*						0.12	Y	Neither
*Haldea striatula*	*Carphophis amoenus*						0.10	Y	Neither

### Second Test Dataset (TD2)

In our second test dataset (TD2, containing images for all 45 classes taken from iNaturalist after the challenge had started), F1 = 0.83 and log-loss = 0.66 (Table 1). Species classification accuracy ranged from 97% for *Pantherophis guttatus* to 61% for *Pantherophis alleghaniensis*. Interclass confusion was 0 for 1118/2025 (55%) of class pairs. Again, the most frequently confused were members of the *Pantherophis obsoletus* complex (Table 2).

Among species that are uncontroversially delimited, there were five species pairs with confusion >10% in TD2 (Table 3). The highest confusion among species in different genera was *Pituophis catenifer* as *Lichanura trivirgata* (14%). As in TD1, *Pituophis catenifer* and *Lichanura trivirgata* co-occur in the Sonoran Desert and are probably frequently photographed against similar backgrounds, although they are dissimilar in appearance. The highest confusion among species/genera found on different continents in TD2 was *Coluber constrictor* as *Hierophis viridiflavus* (7%). Both are slender, fast-moving, diurnal snakes, and the subspecies *H. v. carbonarius* (sometimes considered a full species; Mezzasalma et al., 2015) from Italy is all black, making it very similar in appearance to adult forms of *C. c. constrictor* and *C. c. priapus* in the eastern United States.

Species pairs that were commonly confused in both TD1 and TD2 were *Coluber constrictor* as *Pantherophis alleghaniensis* (9%; also confused 6% of the time in TD1), *Opheodrys vernalis* as *Opheodrys aestivus* (8%; also confused 23% of the time in TD1) and *Nerodia fasciata* as *Nerodia erythrogaster* (7%; also confused 11% of the time in TD1).

### Third Test Dataset (TD3)

In our third test dataset (TD3, containing images for just 27 of the 45 classes hand-selected from other sources), F1 = 0.53 and log-loss = 1.19 when all 45 classes were considered (TD3a), and F1 = 0.73 and log-loss = 1.03 when only the 27 classes relevant to both datasets were considered (TD3b; Table 1). In TD3, species classification accuracy ranged from 100% for 10 species to 25% for *Nerodia erythrogaster*. Interclass confusion was 0 for 648/729 (89%) of class pairs.

Among species that are uncontroversially delimited, there were 23 pairs with confusion >10% in TD3 (Table 3), including two that were also commonly confused in both TD1 and TD2 (*Opheodrys vernalis* as *Opheodrys aestivus*; 33% in TD3, 8% in TD2, 23% in TD1; *Nerodia fasciata* as *Nerodia erythrogaster*; 25% in TD3, 7% in TD2, 11% in TD1) and one that was also commonly confused in TD1 (*Storeria occipitomaculata* as *Storeria dekayi*; 18% in TD3 vs 10% in TD1). The highest confusion among species in different genera was 25% for two species pairs (*Heterodon platirhinos* as *Nerodia erythrogaster* and *Storeria occipitomaculata* as *Nerodia erythrogaster*) and the highest confusion among species that do not occur in sympatry was *Charina bottae* as *Haldea striatula* (22%) (see Table 3 for detailed comparison).

When we asked human experts to identify the same images, they performed slightly better overall (species-level F1 = 0.76 for 45 classes, F1 = 0.79 for 27 classes; Table 1), with significant variation among species (Durso et al., 2021). Humans correctly labeled the 248 images in TD3 at the species level 53% of the time (N = 26,672), although this varied by species from 83% for *Agkistrodon contortrix* to 30% for *Nerodia erythrogaster*.

Thirteen species pairs were confused >10% of the time by humans, five of which also exceeded 10% in at least one of the three test datasets and all but one of which involved species that were also commonly confused by the algorithm (Table 3). Among frequently confused species from TD1 and TD2, humans only had the option to select “Black Ratsnake complex”, so there were no opportunities for confusion among the three putative species (*P. obsoletus*, *P. alleghaniensis*, and *P. spiloides*). Recent changes to taxonomy resulting in unfamiliar names may also hinder human experts’ ability to identify snakes (Carrasco et al., 2016), which could partially explain why e.g., *Haldea striatula* (more widely known as *Virginia striatula* in recent decades; Powell et al., 1994; McVay and Carstens, 2013) was correctly identified to the species level by humans only 31% of the time, or why *Lampropeltis californiae* was identified as *Lampropeltis getula* (the species from which it was split; Pyron and Burbrink, 2009) 23% of the time.

Some of the images in this dataset were purposefully chosen to be as difficult to identify as possible—e.g., low resolution, out of focus, with the snake filling only a small part of the frame, and/or obscured by vegetation. Stallkamp et al. (2012) also found that humans were superior to computer vision at identifying images with visual artifacts (e.g., traffic signs with graffiti or a lot of glare).

### Patterns Across Test Datasets

Because TD1 is a withheld subset of the training data and TD2 was collected using very similar methods, their similarity to the training data is very high. In contrast, TD3 images were selected to be representative of realistic difficult cases. We suggest that this explains the drop in performance for TD3, because image datasets often have built-in biases that are difficult to pinpoint and tests of cross-dataset generalization are rare (Dollár et al., 2009) but generally show that algorithms perform better on their “native” test sets (here, TD1 and TD2) than on entirely novel testing datasets (here, TD3) (Torralba and Efros, 2011). Of the types of bias discussed by Torralba and Efros (2011), we took pains to eliminate or account for label bias (inconsistent category definitions across datasets) by using the same snake taxonomy consistently and paying particular attention to cases where instability in species definitions might sabotage identification predictions. Our dataset is probably most susceptible to capture bias (photographers tending to take pictures of objects in similar ways) and perhaps to selection bias (source datasets that prefer particular kinds of images). Future studies should address the trade-offs between effort and information gain that may result from attempting to acquire images taken from particular angles or of particular anatomical features (Rzanny et al., 2017; Rzanny et al., 2019).

**FIGURE 6:**
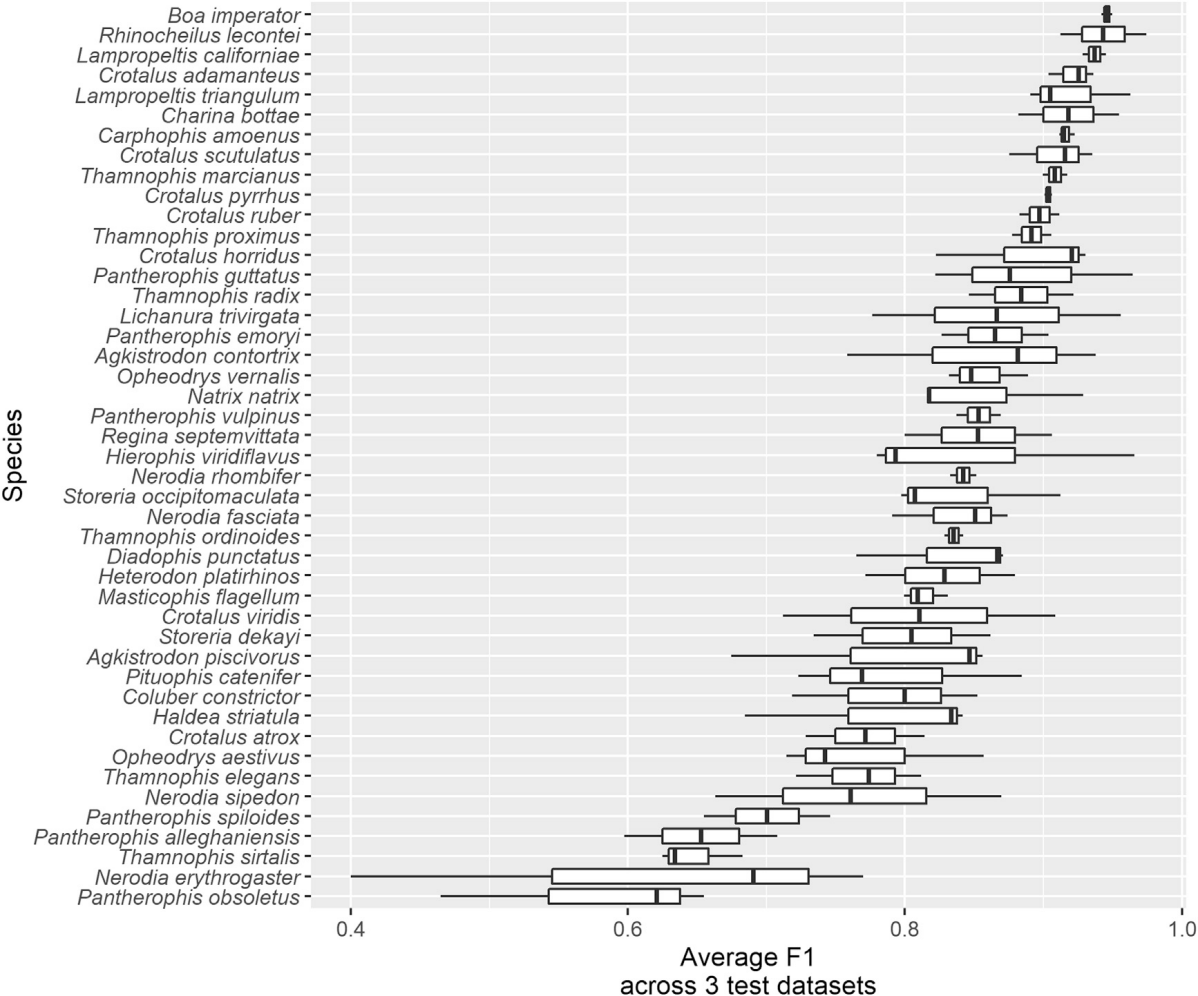
Average F1 across three test datasets for all 45 species.

Species that were consistently identified with high accuracy across the three test datasets include:

• *Boa constrictor* (Boa Constrictor) had the highest average F1 (Figure 6), the fourth highest average recall (Supplementary Figure S3) and the third highest average precision (Supplementary Figure S4), as well as among the lowest average false positive (6.3 ± 2.7%) and average false negative (4.0 ± 2.3%) rates, across the three test datasets. These large, iconic snakes are easily recognized and there are few similar species with which they could be confused, especially within their range. Potential for confusion with large *Python* and *Eunectes* (anaconda) species exists. Several recent studies have suggested that *Boa constrictor* is likely to be a species complex containing as many as nine species (see Reynolds and Henderson, 2018 for a summary).
• *Lichanura trivirgata* (Rosy Boa) had the highest average recall (true positive rate) across three test datasets (95.7 ± 3.3%; Supplementary Figure S3). These short, stocky snakes are native to southwestern North America and are quite distinct in shape within their range; they most closely resemble species of *Eryx* (sand boas) from the Middle East and south Asia, which were not represented in our dataset. However, this species had only moderate average precision; in particular, it was confused with other species that are often photographed against similar backgrounds (e.g., *Crotalus atrox* in TD1, *Pituophis catenifer* in TD2).
• *Pantherophis guttatus* (Red Cornsnake) had the highest average precision (positive predictive value) across three test datasets (98.1 ± 1.6%; Supplementary Figure S4). These snakes are native to the southeastern United States and are common in the pet trade. Our dataset may contain more images of captive *P. guttatus* than any other species, although we attempted to filter these out. The impact of incorporating images of captive snakes in training data intended to be used for identifying wild snakes is not well understood, but the plethora of designer color morphs that have been produced by captive breeding of cornsnakes probably influences the algorithm’s ability to recognize this species.
• *Rhinocheilus lecontei* (Long-nosed Snake) had the second-highest average precision (97.2 ± 0.6%; Supplementary Figure S4) and second-highest average F1 (94.3 ± 4.4%; Figure 6) and relatively high average recall (91.8 ± 8.8%; Supplementary Figure S3). This species is placed in its own monotypic genus, but it is part of a mimicry complex including its non-venomous relatives in the genera *Lampropeltis* (kingsnakes and milksnakes) and *Cemophora* (Scarletsnake) (as well as many other mimics in Central and South America) and their dangerous models in the genera *Micrurus* and *Micruroides* (coralsnakes) (Savage and Slowinski, 1992; Davis Rabosky et al., 2016). The mimicry of *Rhinocheilus* is not as exact as that of many species, but addition of similar species to the training dataset would certainly increase the computer vision algorithm’s confusion of species that currently have low confusion (e.g., *Rhinocheilus lecontei* could be confused with several *Lampropeltis* species on which the algorithm is not currently trained; Shannon and Humphery, 1963; Manier, 2004).

Species groups that were consistently confused across the three test datasets include:

• The two greensnake species in the genus *Opheodrys*: *O. aestivus* (Rough Greensnake) and *O. vernalis* (Smooth Greensnake; formerly placed in the genus *Liochlorophis*). Average F1 across test datasets and species was 81.4 ± 6.9% (Figure 6). These sister taxa are similar in their bright green color, slender bodies, small size, and gestalt; they differ anatomically mainly in that *O. aestivus* has keeled dorsal scales in 17 rows, whereas *O. vernalis* has smooth dorsal scales in 15 rows, subtle characteristics that may not be discernible in photos that are not taken at close range. Habitat and geographic range are also useful in distinguishing them (Ernst and Ernst, 2003). Although telling *Opheodrys* species apart is not likely to ever be clinically or epidemiologically useful, there are many other solid bright green snakes lacking easy-to-distinguish features in Africa, including non-venomous *Philothamnus* and *Hapsidophrys* as well as some highly dangerous *Dispholidus* (boomslangs) and *Dendroaspis* (mambas); see e.g., Broadley and Fitzsimons (1983) and Chippaux and Jackson (2019).
• Watersnake species in the genus *Nerodia*, especially *N. fasciata* (Banded Watersnake), *N. erythrogaster* (Plain-bellied Watersnake), and *N. sipedon* (Northern Watersnake). Average F1 across test datasets and species was 75.9 ± 13.8% (Figure 6). All species in this genus are brown and blotched, but they can be reliably identified using differences in dorsal and ventral pattern, scalation, and range (Gibbons and Dorcas, 2004). However, a photo that does not show the posterior part of the body (where the alignment of blotches is an important characteristic) or the venter (which is commonly concealed in photos) may represent a challenging ID for a human, especially in the absence of geographic information. Again, telling *Nerodia* species apart is not likely to ever be clinically or epidemiologically useful, but they are often confused with *Agkistrodon piscivorus* (Cottonmouth) by laypeople, and worldwide there are many brown, blotched snakes that look similar and may mimic one another (e.g., Gans, 1961; Gans and Latifi, 1973; Kroon, 1975) as well as species that can only be reliably identified if a particular part of the body is visible. Although k nearest-neighbor techniques have been used to semi-automatically classify snakes from images based on their anatomical features (James et al., 2014; James, 2017), these involve laborious and detail-oriented curation of feature databases that should include every possible snake species, a gargantuan task that, although useful for snake taxonomy, would require constant updating (see e.g., Meirte, 1992 for an example of a dichotomous key based on scale characters used by humans). Semi-supervised self-learning algorithms such as ours do not require such databases and represent a more efficient, modern approach to image classification, and wisely chosen pipelines of local feature detection, extraction, encoding, fusion and pooling allows for high accuracy in species classification in groups even more biodiverse than snakes (Seeland et al., 2017).
• The *Pantherophis obsoletus* (North American Ratsnake) species complex (Burbrink, 2001). All three putative species were among the bottom five in terms of F1, never exceeding 74.6% (average across all test datasets and all three species = 63.5 ± 9.0%; Figure 6), as well as the bottom five for recall (Supplementary Figure S3) and the bottom 10 for precision (Supplementary Figure S4). The practice of delimiting species largely on the basis of mitochondrial DNA haplotypes (or combining these with nuclear genetic data sets with little phylogenetic signal) has been criticized for being sensitive to gaps in data caused by limited geographic sampling, which are artifactually detected as species boundaries by clustering algorithms used in the Multispecies Coalescent Model for species delimitation, leading to “over-splitting” of widely distributed species and assignment of species names to “slices” of continuous geographic clines that are not evolving independently (Hillis, 2019; Chambers and Hillis, 2020) and may not be morphologically diagnosable (Guyer et al., 2019; Freitas et al., 2020; Mason et al., 2020). Here we show that computer vision algorithms have low power to distinguish at least some putative species that have been delineated primarily using molecular methods and where training data were identified exclusively from photos, presumably primarily based on geographic location (Table 2). Although Burbrink (2001) provided multivariate analyses of 67 mensural and meristic characters (mostly related to scalation) that corresponded to mitochondrial lineages, the most obvious color and pattern variants intergrade with one another over large areas and correspond to former subspecies designations rather than Burbrink’s species concepts (recently refined to better match phenotypic variation; Burbrink et al., 2020; Hillis and Wüster, 2021), and numerous color patterns can be found within each putative species, especially *P. alleghaniensis* (not to mention the marked ontogenetic change characteristic of all three, wherein juveniles are most similar to adult *P. spiloides* from the southern portion of their range). This, combined with the widely overlapping distribution between *P. alleghaniensis* and *P. spiloides*, makes the three putative species nearly impossible for both humans and AI to differentiate in the absence of information about geographic location. We also note that training images in this species complex are probably more likely to be mis-labeled due to confusion over how to differentiate the putative species.
• Other taxa used in our dataset that may be susceptible to the same problem as the *Pantherophis obsoletus* complex include *Lampropeltis californiae* and *Lampropeltis triangulum* (both members of widespread species complexes that were formerly treated as a single species, of which only one putative species per species complex is present in our dataset; Pyron and Burbrink, 2009; Ruane et al., 2014) and *Agkistrodon contortrix* and *Agkistrodon piscivorus* (both of which have recently been split into two putative species along former subspecies lines but were treated as single species in our dataset; Burbrink and Guiher, 2015) as well as *Boa constrictor* (the taxonomy of which is even less settled; see Reynolds and Henderson, 2018 for a summary). See Appendix I for a full list of species in our dataset together with notes on recent taxonomic changes that may affect their diagnosability based purely on a photograph.

Of the 44 species pairs that exceeded 10% confusion on any test dataset (including human IDs), only four had no overlap in geographic range (two others had very little geographical overlap). Incorporating information on the geography has the potential to better discriminate these species pairs (Wittich et al., 2018), but in the most biodiverse regions of the world, >125 species of snakes may be sympatric within the same 50 × 50 km^2^ area (Roll et al., 2017).

Most commonly confused species pairs involved either two non-venomous or two medically important venomous species (hereafter “venomous”). Of the 44 species pairs that exceeded 10% confusion on any test dataset (including human IDs), 31 involved two non-venomous species and five involved two venomous species (Table 3). Only three of the 44 pairs were “false positives” (a non-venomous species identified as a venomous one) and only five were “false negatives” (a venomous species identified as a non-venomous one). Bites from all medically important venomous snakes in our dataset (all North American pit vipers) would be treated using the same antivenom (Dart et al., 2001; Gerardo et al., 2017) but this would not be as straightforward in other regions of the world (e.g., *Bitis* vs. *Echis* in sub-Saharan Africa) and indeed is becoming more complex in North America as new products enter the market (Bush et al., 2015; Cocchio et al., 2020).

**FIGURE 7:**
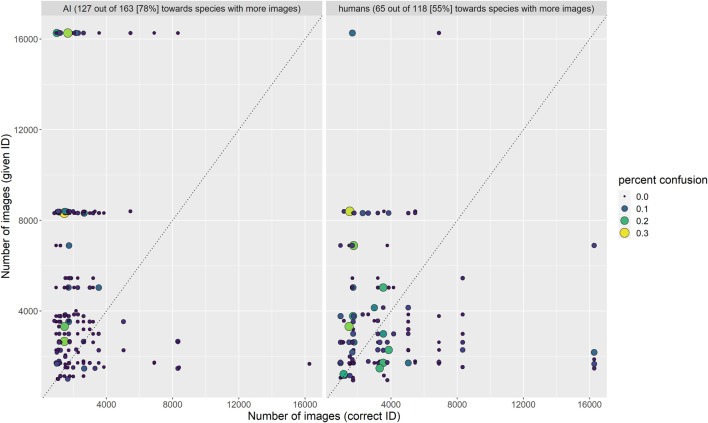
Frequency with which confused species pairs were incorrectly identified by algorithm and humans. The algorithm erred on the side of the species with more training images much more often than humans.

Finally, we found that among the 163 confused species pairs in TD3, the algorithm suggested the species with more images in the training dataset as the identity of the species with fewer images far more often (78% of pairs) than the other way around. For example, *Opheodrys vernalis* (1,471 training images) was misidentified as *O. aestivus* (3,312 training images) 33% of the time in TD3, but *O. aestivus* was never misidentified as *O. vernalis* in TD3, even though humans confused these two species nearly equally often in both directions (12 and 10%). Humans were marginally more likely to suggest the species from a given pair with more images even when it was incorrect (65 of 118 confused pairs; 55%), but the effect was much more pronounced for AI than for humans (Figure 7). For the algorithm, this is probably due to the imbalance in the amount of training data among classes; for humans, we suggest that it may be caused by the same processes that lead a species to be represented by fewer images in the training data: smaller geographic ranges or lower probabilities of encounter, leading to less familiarity with the rarer species.

Long-tailed distributions are commonplace and have been called “the enemy of machine learning” (Bengio, 2015). As we enlarge our dataset to include more of the snake species with few images per species (i.e., those in the “long-tailed” part of the distribution), we must pay particular attention to rare species. Previous studies on faces (Zhang et al., 2017) and scene parsing (Yang et al., 2014) suggest approaches including rare class enrichment (Yang et al., 2014) and clustering objects into visually similar hierarchical groups (in our case, snake genera or families) (Ouyang et al., 2016).

## Conclusion

It is extremely important to keep in mind that the 45 species that the algorithm was trained on were selected solely based on the quantity of photos available, and no effort was made to include all possible similar or closely related species. Additionally, human experts could suggest any of the >3,700 species of snakes as an identification, and species other than the 45 used to train the computer vision algorithm were suggested (always incorrectly) 76% of the time overall (min = 7% for *Agkistrodon contortrix*, max = 50% for *Hierophis viridiflavus*). The average number of species that a human expert knows well (analogous to the number of classes that an algorithm is trained on) is hard to estimate. The grand total number of species suggested by all 580 human experts was 457, but the mean ± SD per person was just 35 ± 18 (min = 18, max = 61; Durso et al., 2021). Another important difference was that human experts had access to the geographic location of each image, whereas the computer vision algorithm does not yet incorporate this information. To further explore these issues, additional rounds of our AICrowd challenge, including more photos, additional species classes, and information on geography at the continent and country level have allowed us to assess the generality of patterns discovered here (Bloch et al., 2020; Moorthy, 2020; Picek et al., 2020). Future directions include finer image segmentation (e.g., the head, body, and tail of the snake) and hierarchical (e.g., genus level) classification, which might work better when more species from larger genera are included.

The accuracy of our algorithm at predicting species-level identity ranged from 72 to 87% depending on the test dataset (Table 1), which is comparable to that for other taxa (see Table 2 of Weinstein, 2018). A promising future awaits snake identification as AI begins to compliment static photos, diagrams, and audio-visual media, interactive multiple-access keys, species checklists that can be customized to particular locations, dynamic range maps, and online communities in which people share species observations and identifications in “next-generation field guides” (Farnsworth et al., 2013). In particular, we emphasize the need to keep “humans in the loop” in order to validate labels in training datasets as well as AI predictions, particularly for healthcare applications (Holzinger, 2016; Holzinger et al., 2016).

We suggest that AI could play a larger role in disease surveillance and disease ecology (Hosny and Aerts, 2019; Pandit and Han, 2020), particularly as a widely available rapid diagnostic test for improving detailed epidemiological reporting and mapping of snakebite, as well as clinical management (Ruiz De Castañeda et al., 2019). However, a more complete understanding of how AI and humans perform and interact with one another when identifying species classes from photos with regard to image quality, inter-species similarity, and prior information about geographic location is essential before this technology could aid in the snakebite crisis. Critically, increasing data availability in regions of high snake diversity and snakebite prevalence is a pre-requisite for applying data-hungry algorithms to the regions of the world where they are most needed.

## Data Availability

The raw data supporting the conclusions of this article will be made available by the authors, without undue reservation.
