# Supplementary figures and images for: Supervised Learning Computer Vision Benchmark for Snake Species Identification From Photographs: Implications for Herpetology and Global Health
Source: Front Artif Intell. 2021 Apr 20;4:582110. doi: 10.3389/frai.2021.582110 (PMC8093445; doi:10.3389/frai.2021.582110)

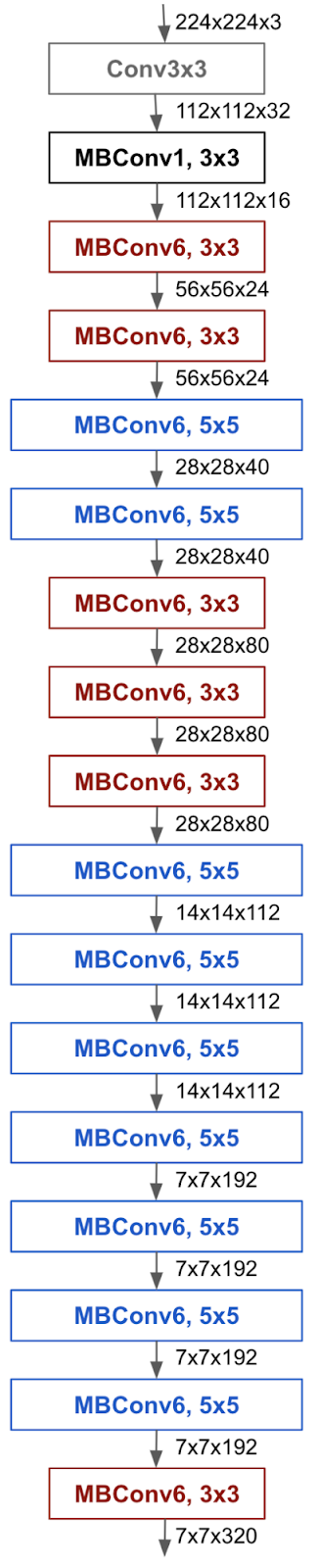

Supplement: Supplementary file 1 [file Image1.TIF]

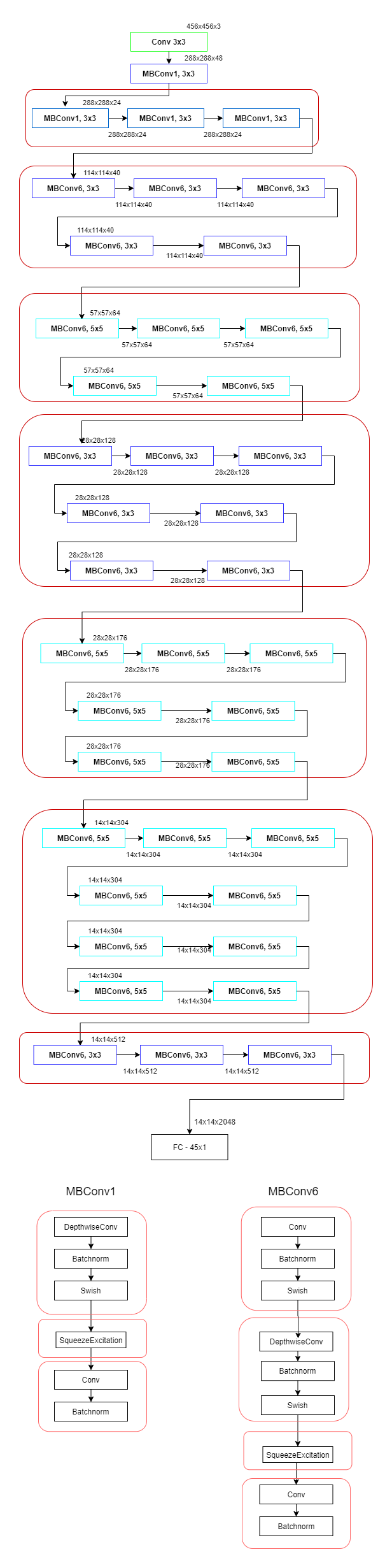

Supplement: Supplementary file 2 [file Image2.TIF]

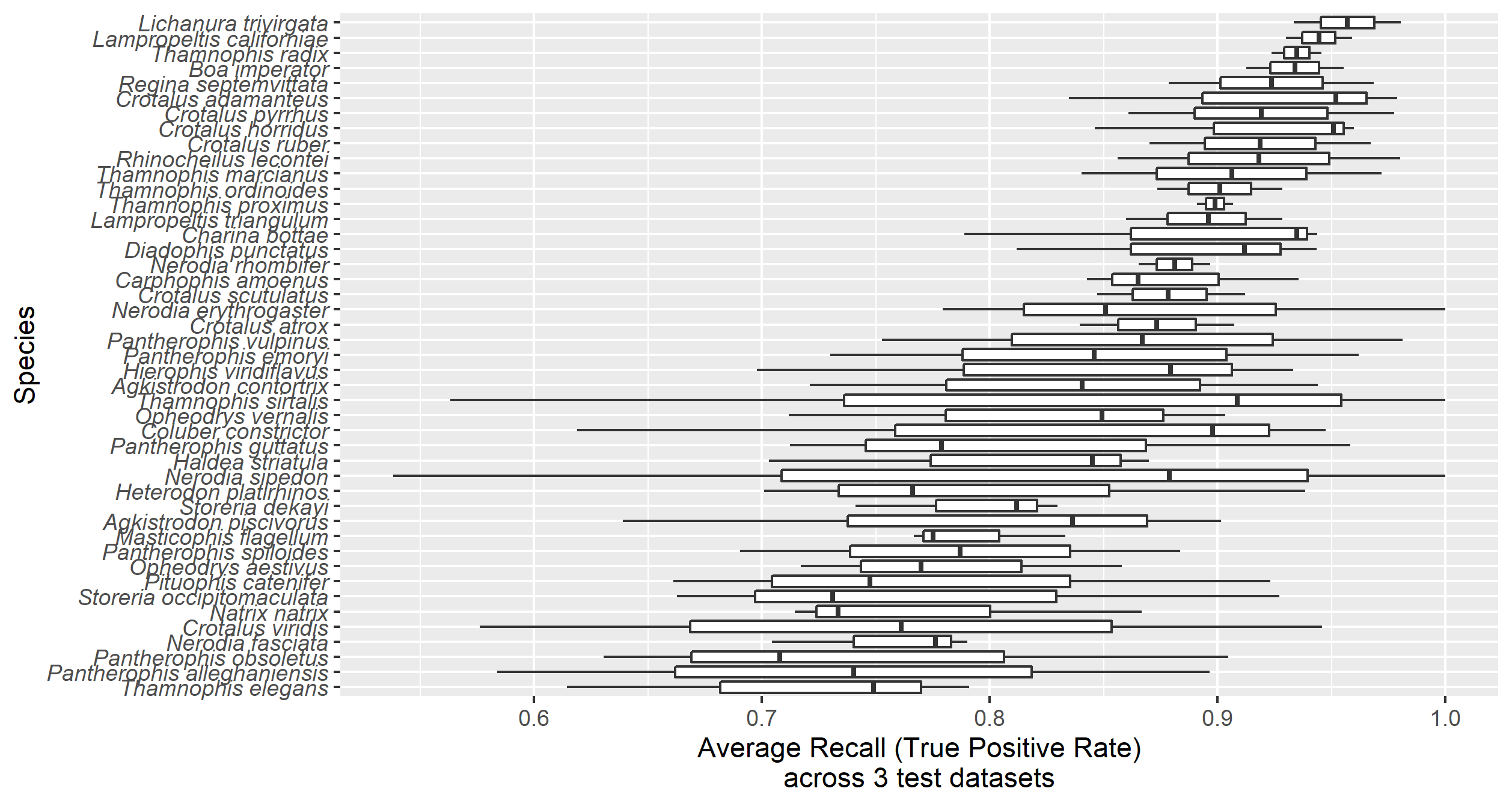

Supplement: Supplementary file 3 [file Image3.TIF]

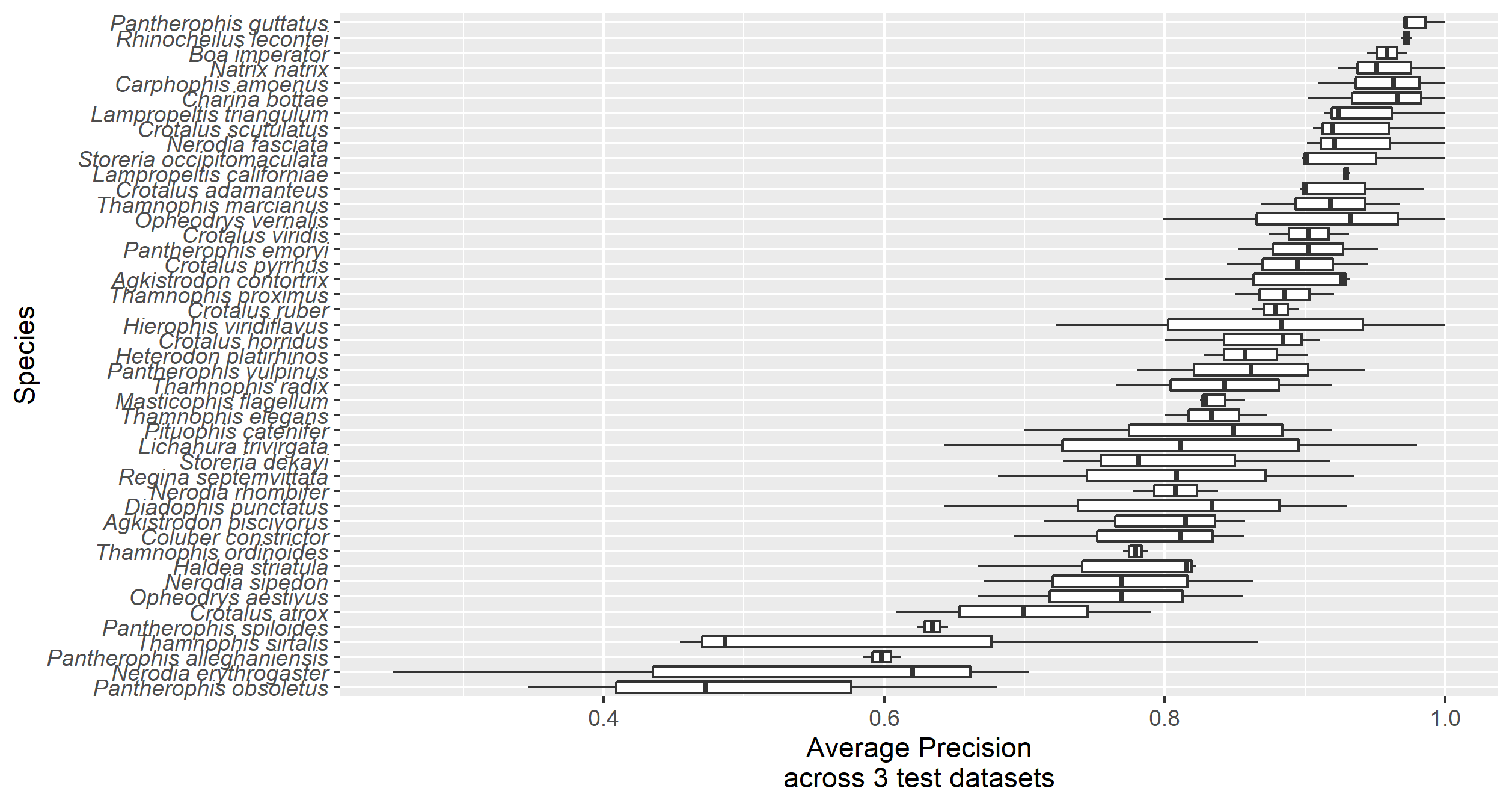

Supplement: Supplementary file 4 [file Image4.TIF]
